# Footwear Toe‐Box Shape and Medial Forefoot Pressures in Women With Hallux Valgus

**DOI:** 10.1002/jfa2.70041

**Published:** 2025-04-12

**Authors:** Katrina J. Bajraszewski, Polly Q. X. Lim, Andrew K. Buldt, Sheree E. Hurn, Karen J. Mickle, Edward Roddy, Anita E. Wluka, Bircan Erbas, Shannon E. Munteanu, Hylton B. Menz

**Affiliations:** ^1^ Discipline of Podiatry School of Allied Health Human Services and Sport La Trobe University Bundoora Australia; ^2^ School of Clinical Sciences Faculty of Health Queensland University of Technology Kelvin Grove Australia; ^3^ Applied Sport Science and Exercise Testing Laboratory College of Health Medicine and Wellbeing University of Newcastle Ourimbah Australia; ^4^ School of Medicine Keele University Keele UK; ^5^ Haywood Academic Rheumatology Centre Midlands Partnership University NHS Foundation Trust Haywood Hospital Burslem UK; ^6^ School of Public Health and Preventive Medicine Monash University Victoria Australia; ^7^ School of Psychology and Public Health La Trobe University Victoria Australia

**Keywords:** biomechanics, forefoot, hallux valgus, human, pressure, shoes

## Abstract

**Background:**

Narrow fitting footwear is a modifiable risk factor for the development of hallux valgus (HV). Despite this, the pressure that footwear exerts at the medial forefoot has not been fully evaluated in people with HV. Therefore, the objective of this study was to determine whether the toe box of footwear habitually worn by women with HV is associated with pressure exerted on the medial forefoot.

**Methods:**

In‐shoe peak pressure and maximum force at the medial forefoot (distal and proximal sites) were recorded from 28 women (mean age 60.7 years, SD 10.7) with moderate or severe HV using the pedar pad pressure system (Novel GmbH, Germany). The shape (width and area) of the participants' most symptomatic foot and toe‐box of their usual footwear was determined using an INFOOT 3D laser scanner (I‐Ware Laboratory, Japan) and hand tracing, respectively. The difference between the foot and corresponding footwear measurements as well as differences in the magnitude and timing of peak pressure and maximum force between the proximal and distal forefoot were determined using independent *t*‐tests. Correlations between forefoot pressures with toe‐box differential were determined using Spearman's *ρ* analyses.

**Results:**

Peak pressure and maximum force were significantly greater (mean difference [MD] = 33.0 ± 15.4 kPa; *p* < 0.001 and 12.8 ± 7.3 N; *p* = 0.001) and occurred slightly later in the stance phase at the distal forefoot compared to the proximal forefoot (MD = 6.0 ± 6.9%; *p* = 0.083 and 6.9 ± 6.8%; *p* = 0.045, respectively). There were no significant correlations between toe‐box differential and medial forefoot pressures, with all correlations less than 0.35 (*p* > 0.05).

**Conclusion:**

Toe‐box shape and fit of footwear typically worn by older women with painful HV was not associated with increased medial forefoot pressures in this study sample. Therefore, changing the toe‐box width and area of the usual footwear worn by older women with painful, moderate or severe HV may not necessarily reduce medial forefoot pressures where footwear does not appear to play a role.

## 1 Introduction

Hallux valgus (HV) is a common foot condition characterised by the medial deviation of the first metatarsal and lateral deviation of the hallux, resulting in progressive subluxation of the first metatarsophalangeal joint and a medial prominence of the forefoot [1]. The prevalence of HV is greater among females and is estimated at 23% in adults and 36% in older adults (aged over 65 years), indicating an increase with age [2]. HV is associated with reduced hallux strength [3, 4], impaired balance [5, 6] and increased risk of falls [7, 8]. Both general and foot‐related quality of life progressively decline with increasing HV severity [9, 10].

Ill‐fitting footwear is a potentially modifiable risk factor for HV [11, 12]. Between 63% and 72% of people wear inappropriately sized footwear and are consequently more likely to have foot pain and foot disorders [13]. The primary footwear feature implicated in HV development is a narrow toe box (the section of footwear that surrounds the toes), particularly among women [14, 15]. Moreover, among women, the development of HV is significantly associated with the use of a narrow toe box between the ages of 20–39 years [11]. This suggests that footwear commonly worn by people who develop HV does not follow the contour of the forefoot and that the toes may adapt to footwear shape, thereby contributing to the onset and progression of the deformity.

Despite several studies examining plantar pressures associated with HV [16], only one study has investigated the effect of the toe‐box shape on medial forefoot pressures in women with HV [17]. This study reported no significant difference in peak pressures at the medial first metatarsophalangeal and interphalangeal joint with round toe‐box footwear compared to participants' own footwear. However, the shape of participants' own footwear was not reported in this study. Two studies have investigated medial forefoot pressures among other populations and, in contrast, found a relationship between the toe‐box shape and medial forefoot pressures [18, 19]. Among women without HV, peak pressures at the medial first metatarsophalangeal and interphalangeal joints were significantly reduced when wearing footwear with a round toe box compared to a square or pointed toe box [19]. Similarly, among both men and women with foot pain, peak pressures at the medial first metatarsophalangeal joint were significantly reduced when wearing footwear with a round, wide toe box compared to participants' own footwear [18].

The contrast between populations with and without HV indicates that there is still little understanding of the relationship between toe‐box shape and medial forefoot pressures in HV, although individuals with HV are known to have difficulty with finding comfortable footwear [4]. In addition, all three studies collected pressure data by securing discrete sensors to predetermined locations on the foot with an adhesive tape [17, 18, 19]. Limitations of this method of data collection are the possibility of sensor placement error [20] and movement of the sensor during data collection.

Evidence for a relationship between toe‐box shape and fit and medial forefoot pressures in HV is limited and suggests that further investigation is necessary to better understand interventions for the prevention and management of HV pain and deformity. The objectives of this study were therefore to (i) quantify in‐shoe pressures at the distal and proximal medial forefoot (corresponding to the first interphalangeal and metatarsophalangeal joints) in women with HV using a novel matrix pressure sensor and (ii) examine the association between these pressures and forefoot toe‐box differential. We hypothesised that there would be a positive association between first interphalangeal and metatarsophalangeal joint pressures and forefoot toe‐box differential, indicating that the wearing of relatively narrow shoes would increase medial pressures.

## 2 Methods

### 2.1 Study Design

This cross‐sectional study was nested within a randomised feasibility trial for the effectiveness of a multifaceted non‐surgical intervention for HV. The data were collected during the baseline assessment for this trial. A protocol of the trial and the main findings have been published [21, 22, 23]. Reporting of this study followed the Strengthening the Reporting of Observational Studies in Epidemiology guidelines [24]. Ethical approval was granted by the La Trobe University Human Ethics Committee (reference HEC20474). Informed written consent was obtained from all participants. The feasibility trial was registered with the Australian and New Zealand Clinical Trial Registry (ACTRN12621000645853).

### 2.2 Participants

Participants were recruited using posters in the local community, email correspondence to staff of the La Trobe University School of Allied Health, Human Services and Sport, postal invitation to patients of the La Trobe University Health Sciences Clinic and Facebook advertising targeting women in the local area with painful HV and offering treatment free of charge. Inclusion criteria were (i) female, (ii) ≥ 40 years of age, (iii) first metatarsophalangeal or interphalangeal joint pain rated three out of 10 on a zero to 10‐point scale for at least 12 weeks, (iv) HV scored two or more on the Manchester scale [25] equating to moderate or severe HV, (v) capability to ambulate household distances greater than 50m without walking aid and (vi) ability to understand verbal and written English. Exclusion criteria were a history of (i) HV surgery, (ii) neurological disease impacting ambulation, (iii) inflammatory rheumatological conditions (e.g., gout, psoriatic arthritis and rheumatoid arthritis), (iv) lower limb or partial foot amputation, (v) lower limb or back injury that interferes with reaching the feet or (vi) treatment with arch‐contouring orthoses or foot exercises (stretching, mobilisation and strengthening) in the last 12 weeks. The decision to recruit solely women was due to their higher prevalence of HV [2] and tendency to wear narrow footwear [13].

### 2.3 Sample Size

The sample size was determined by the feasibility trial, which did not require full power to detect statistically significant differences between groups. The recommended sample size for feasibility and pilot trials is 12 participants per group [26]. Twenty‐eight participants were recruited to account for a 15% drop‐out rate. The sample of 28 was sufficient to detect a correlation between toe box shape and fit and medial pressures of at least 0.22 (significance level 0.05, power 0.80) [27].

### 2.4 Participant Characteristics and Clinical Assessments

A structured interview was conducted to collect data on participant demographic characteristics (age, education level, ethnicity, primary medical conditions and medications used), anthropometrics (height, weight and body mass index [BMI]), general health and foot health (Short Form 12 questionnaire [28], Incidental and Planned Exercise Questionnaire [29] and the Manchester–Oxford Foot Questionnaire) [30] and clinical features (severity of HV deformity, side affected, pain duration and pain severity) [21, 22].

### 2.5 Foot Shape Assessment

To document foot dimensions, an INFOOT 3D laser scanner (I‐Ware Laboratory, Japan) was used. Scans were taken in a weight‐bearing position, with the weight evenly distributed between each foot. Participants could use an upright bar for support if required. The INFOOT 3D laser scanner has been validated against radiographs and clinical measurements and found to be reliable for the majority of parameters [28]. From these scans, an outline of participants' feet was produced as a vector image. Forefoot width was defined as the length of a line connecting the most medial point of the first metatarsophalangeal joint and the most lateral point of the fifth metatarsophalangeal joint and forefoot area was defined as the area created by the footprint from this line to the distal end of the longest digit.

### 2.6 Footwear Shape Assessment

**FIGURE 1:**
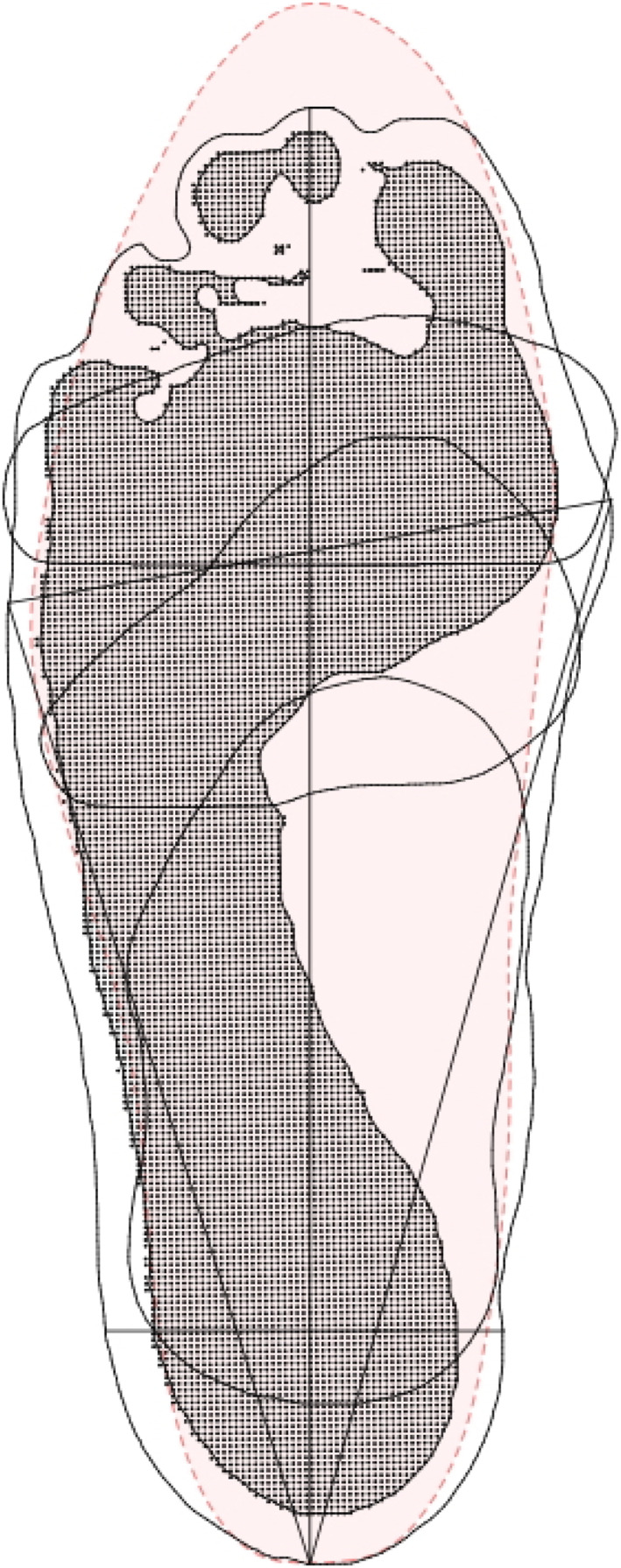
Three‐dimensional foot scan superimposed with a shoe scan. See text for explanation.

Participants were asked to bring the closed‐toe footwear they wore most often. To document toe‐box shape, an outline of each shoe's sole was traced onto blank paper and scanned in a flat‐bed scanner. If the outsole was appreciably wider than the upper of the shoe, the outline was adjusted to approximate the true upper dimensions using a visual estimate after tracing was completed. This method has been successfully used in a previous study [15]. Toe‐box width was defined as the length of a line connecting the most medial point of the forefoot of the shoe and the most lateral point of the forefoot of the shoe, and the toe‐box area was defined as the area created by the outline of the shoe from this line to the distal end of the shoe. To compare foot and footwear shapes, a vector image of the participants' footwear was superimposed over a vector image of the participant's foot using Canvas 11 (ACD Systems, Miami, FL, USA). See Figure 1. To quantify the difference in shape (referred to as the toe‐box differential), two measurements were taken: first, the difference in width at the level of the metatarsophalangeal joints (hereafter referred to as forefoot width differential); second, the difference in area taken between the level of the metatarsophalangeal joints and the distal end of the longest digit (hereafter referred to as forefoot area differential).

### 2.7 In‐Shoe Pressure Assessment

**FIGURE 2:**
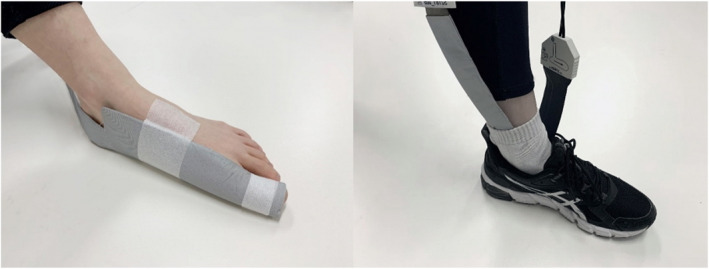
Pedar pad placement in footwear during walking trials.

In this study, we defined ‘medial forefoot’ as two masks corresponding to the first metatarsophalangeal and interphalangeal joints and measured these using the pedar pad (Novel GmbH, Munich, Germany), a flexible strap of 56 sensors with an active area of 70 mm × 160 mm and resolution of 0.5 sensors/cm^2^. The pedar pad is a novel device but uses the same pressure distribution measuring system as the pedar‐X, which has previously been found to be a valid, reliable and accurate measure of in‐shoe pressures [31]. Once the strap was secured to the foot as shown in Figure 2, and the participant's shoes were fastened, the pedar pad was zeroed by asking participants to raise their foot. Participants then completed four 6‐m walking trials at a self‐selected speed. Following the trials, it was checked that the pedar pad remained in place. If the participant had bilateral HV, the foot with the greater symptomatic severity was used. To minimise the confounding effect of variability in walking speed on the pressure data, a trial was repeated if the walking speed differed by more than 5% of the original trial. The middle four steps from each trial (i.e., a total of 16 steps) were used for analysis to prevent acceleration and deceleration from influencing the data. Sixteen steps has been shown to provide reliable measurements [31]. Plantar pressure data were averaged within the pedar pad Novel scientific software, and peak pressure (defined as the highest pressure value recorded in a mask region, measured in kPa) and maximum force (defined as the highest force value recorded in a mask region, measured in N) were documented. The sensor area was divided into two mask regions: mask 1 (distal forefoot, which included two rows of sensors and corresponded to the first interphalangeal joint) and mask 2 (proximal forefoot, which included three rows of sensors and corresponded to the first metatarsophalangeal joint).

### 2.8 Statistical Analysis

Statistical analysis was undertaken using IBM SPSS Statistics version 26.0 (IBM Corp, NY, USA). All data were explored for normality. Differences between distal and proximal peak pressure and maximum force were compared using independent samples *t*‐tests. Associations of distal and proximal forefoot peak pressure and maximum force with the forefoot width and area differential were investigated using the Spearman's rho (*ρ*) correlation coefficient. The following interpretation of correlations was used: 0 to 0.25 indicated little or no relationship, > 0.25 to 0.50 indicated a fair relationship, > 0.50 to 0.75 indicated a moderate to good relationship and > 0.75 indicated a good to excellent relationship [32]. In all analyses, *p* < 0.05 was considered statistically significant. Individual peak pressure and maximum force recordings were time‐normalised using code written in R software incorporating linear interpolation and then graphed using Canvas 11 (ACD Systems, Miami, FL, USA) to analyse timing over the stance phase.

## 3 Results

### 3.1 Participant Characteristics

A total of 28 participants were recruited for the pilot and feasibility trial and all were included in this study. Participant characteristics are shown in Table 1. The group had a mean (SD) age of 60.7 (10.7) years and BMI of 27.2 (4.2) kg/m^2^. The most frequent ethnicity of participants was Oceanic/Australian (*n* = 13, 46%). Most participants displayed bilateral HV (*n* = 23, 82%) and at least moderate deformity (moderate *n* = 19, 68%; severe *n* = 9, 32%). The sample was physically active according to their responses to the Incidental and Planned Exercise Questionnaire.

**TABLE 1:** Participant demographic and clinical characteristics. Values are *n* (%) unless otherwise stated.

Participant characteristic	Value
Demographics and anthropometrics	
Age, mean (SD) [range] years	60.7 (10.7) [44–80]
Height, mean (SD) cm	160.1 (6.9)
Weight, mean (SD) kg	69.8 (11.3)
Body mass index, mean (SD) kg/m^2^	27.2 (4.2)
Education level	
Less than primary school	1 (3.6)
High school (or equivalent) completed	8 (28.6)
TAFE completed	7 (25.0)
College/university completed	6 (21.4)
Post graduate degree completed	6 (21.4)
Ethnicity	
Anglo‐Indian	2 (7.1)
North African and Middle Eastern	4 (14.3)
North‐West European	1 (3.6)
Oceanic (Australian)	13 (46.4)
South‐East Asian	2 (7.1)
Southern and Central Asian	2 (7.1)
Southern and Eastern European	2 (7.1)
Sub‐Saharan African	1 (3.6)
United Kingdom	1 (3.6)
Medical conditions	
Hypertension	9 (32.1)
Osteoarthritis	9 (32.1)
Heart disease	3 (10.7)
Cancer	2 (7.1)
Leg ulcers	1 (3.6)
Use of 4+ medications	6 (21.4)
General health	
SF‐12 scores, mean (SD)^a^	
Physical	44.8 (11.0)
Mental	48.7 (8.9)
Total physical activity (IPEQ), mean (SD) hours/week	40.30 (21.2)
MOXFQ, mean (SD)^b^	
Walking/standing	40.6 (28.2)
Pain	55.0 (20.5)
Social interaction	41.3 (25.4)
MOXFQ‐Index	45.3 (22.3)
Clinical features of HV	
Side affected: Left/right /both	1 (3.6)/4 (14.3)/23 (82.1)
Index foot: Left/right	10 (35.7)/18 (64.3)
Manchester scale for the index foot	
Grade 3 (moderate)	19 (67.9)
Grade 4 (severe)	9 (32.1)
HV pain for index foot, mean (SD)^c^	65.3 (19.6)
HV pain duration for index foot, mean (SD) weeks	60.4 (493.7)

Abbreviations: HV, hallux valgus; IPEQ, incidental and planned exercise questionnaire; TAFE, technical and further education.

^a^ Short Form 12 Health Survey (SF‐12): scores ranged from 0 to 100, with higher scores indicating better function.

^b^ Manchester–Oxford Foot Questionnaire (MOXFQ): scores range from 0 to 4 for each domain, with 4 indicating greater pain and for the MOXFQ‐Index scores, score ranges from 0 to 100, 100 indicating greater pain.

^c^ Visual analogue scale (VAS): scores ranged from 0 to 100, with higher scores indicating worse pain.

### 3.2 Foot and Footwear Characteristics

Forefoot width was greater than toe‐box width in 10 cases (36%) and narrower in 18 cases (64%). The mean (SD) forefoot width was 102.4 mm (6.0) and the mean (SD) toe‐box width was 99.8 mm (7.9). This difference was not statistically significant (mean difference [MD] = 2.6 mm, 95% CI = −1.1 to 6.4; *p* = 0.168). The forefoot area was smaller than the toe‐box area in 27 cases (96%) and greater in one case (4%). The mean (SD) forefoot area was 42,435 mm^2^ (8861) and the mean (SD) toe‐box area was 56,260 mm^2^ (13,825). This difference was statistically significant (MD = −13,825 mm^2^, 95% CI = −20,047 to −7603 *p* < 0.001).

### 3.3 Pressure and Force Characteristics at the Distal and Proximal Forefoot

**FIGURE 3:**
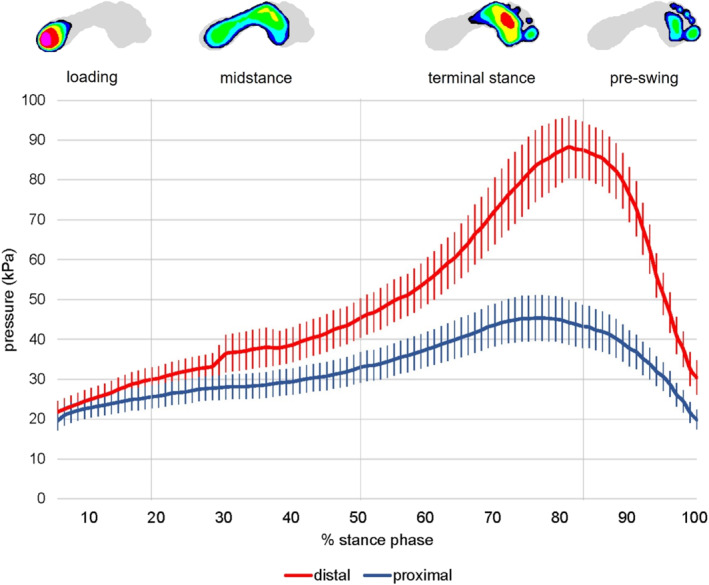
Peak pressure (kPa) at the medial forefoot over stance phase. The following intervals for subphases in stance phase were used: loading (0%–20% of stance), midstance (> 20% to 52%), terminal stance (> 52% to 83%) and pre‐swing (> 83% to 100%).

**FIGURE 4:**
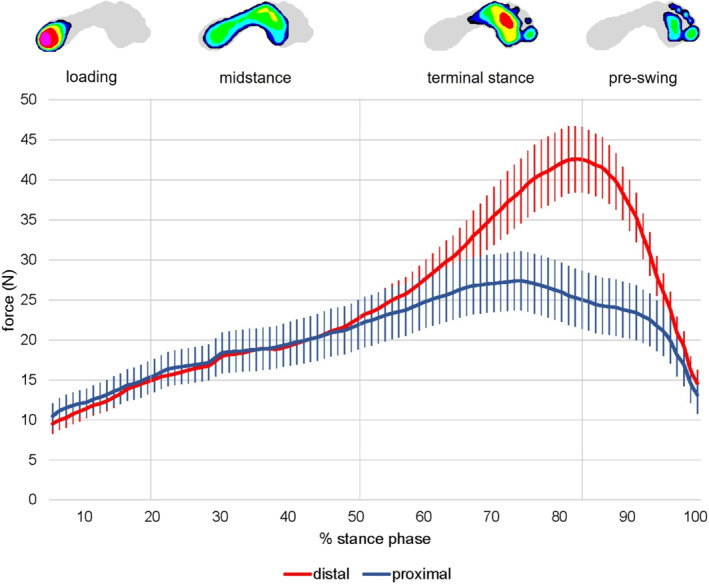
Maximum force (N) at the medial forefoot over stance phase. The following intervals for subphases in the stance phase were used: loading (0%–20% of stance), midstance (> 20% to 52%), terminal stance (> 52% to 83%) and pre‐swing (> 83% to 100%).

Peak pressure and maximum force at the distal and proximal forefoot are shown in Table 2. Peak pressure (MD = 33.0 kPa, 95% CI = 17.7 to 48.4; *p* < 0.001) and maximum force (MD = 12.8 N, 95% CI = 5.5 to 20.1; *p* = 0.001) were significantly greater at the distal compared to proximal forefoot. Peak pressure and maximum force at both the medial forefoot regions over stance phase are shown in Figures 3 and 4, respectively. Time to peak pressure at the proximal and distal forefoot occurred at 73.7% (17.8) and 79.8% (10.2) of stance phase, respectively. This difference was not statistically significant (MD = 6.0%, 95% CI = −0.8 to 12.9; *p* = 0.083). Time to maximum force at the proximal and distal forefoot occurred at 73.3% (17.5) and 80.2% (10.1) of stance phase, respectively. This difference was statistically significant (MD = 6.9%, 95% CI = 0.15 to 13.7; *p* = 0.045).

**TABLE 2:** Peak pressure (kPa) and maximum force (N) at the distal and proximal forefoot and the correlation with forefoot width differential and area differential.

Variable	Region	Mean (SD)	Forefoot width differential^a^ correlation (*p*‐value)	Forefoot area differential^b^ correlation (*p*‐value)
Peak pressure	Distal forefoot	91.32 (41.79)	−0.15 (0.443)	0.13 (0.503)
	Proximal forefoot	58.30 (32.77)	−0.27 (0.172)	0.16 (0.427)
Maximum force	Distal forefoot	43.27 (18.91)	−0.10 (0.634)	0.23 (0.244)
	Proximal forefoot	30.40 (19.36)	−0.08 (0.680)	0.35 (0.065)

^a^ Width differential = difference between the width of the forefoot and the width of the toe box, with a greater/positive value indicating the forefoot is wider.

^b^ Area differential = difference between the area of the forefoot and the area of the toe box, with a greater/positive values indicating the forefoot is larger.

### 3.4 Relationships Between Peak Pressure and Toe‐Box Dimensions

Correlations between peak pressures during walking and measurements of toe‐box dimensions are shown in Table 2. There was little to no relationship between distal forefoot peak pressure and forefoot width differential (*ρ* = −0.15, *p* = 0.443) and a fair nonstatistically significant relationship between proximal forefoot peak pressure and forefoot width differential (*ρ* = −0.27, *p* = 0.172). There was little to no relationship between distal forefoot peak pressure and forefoot area differential (*ρ* = 0.13, *p* = 0.503) and little to no relationship between proximal forefoot peak pressure and forefoot area differential (*ρ* = 0.16, *p* = 0.427).

### 3.5 Relationships Between Maximum Force and Toe‐Box Dimensions

Correlations between maximum force during walking and measurements of toe‐box dimensions are shown in Table 2. There was little to no relationship between distal forefoot maximum force and forefoot width differential (*ρ* = −0.10, *p* = 0.634) or proximal forefoot maximum force and forefoot width differential (*ρ* = −0.08, *p* = 0.680). There was little to no relationship between distal forefoot maximum force and forefoot area differential (*ρ* = 0.23, *p* = 0.244) and a fair nonstatistically significant relationship between proximal forefoot maximum force and forefoot area differential (*ρ* = 0.35, *p* = 0.065).

## 4 Discussion

In this study, we used a novel technique to measure in‐shoe medial forefoot pressures in women with HV, with the aim of determining whether the shape of the toe‐box influences these pressures during walking. We found that peak pressure and maximum force occurred slightly earlier during late stance phase at the proximal forefoot compared to the distal forefoot. However, we found only little to fair, nonstatistically significant relationships between toe‐box dimensions and medial forefoot pressures, suggesting that the two‐dimensional shape of the shoes (as measured using length and area differential) worn by our sample may not influence medial forefoot loading in HV. Interestingly, the shoe toe‐box area was significantly larger than the foot, so it is possible that the shoes worn were not overly constrictive in most of our participants.

Our findings that significantly greater peak pressure and maximum force occurred at the distal forefoot compared to the proximal forefoot are in contrast to previous studies that reported greater peak pressure at the first metatarsophalangeal joint compared to the first interphalangeal joint [18, 19]. This disparity may be due to differences in methods to obtain pressure data. Previous studies used discrete sensors to obtain pressure data, whereas we used a matrix device. The matrix device provides a large area for pressures to be detected, compared to discrete sensors that detect pressure at isolated regions and may move during testing.

Our findings that the relationships between toe‐box shape and medial forefoot pressures were weak and not statistically significant were unexpected, given that narrow‐fitting footwear is considered an important risk factor for the development of HV [11]. In accordance with our findings, the only other study among people with HV found no significant difference in pressures at the medial forefoot between participants own footwear and footwear with a round accommodative toe box [17]. Notably, this study and our own included people with moderate or severe HV (none had no or mild HV), who may self‐select footwear that does not increase pressure on their HV deformity. Indeed, because our sample had symptoms for some time, many had already changed their footwear to make it more comfortable. We also did not control for the compliance of the materials used for the upper of participants' footwear and noted that in some participants, the upper of the shoe was already stretched as a result of their HV. Indeed, a relationship between pressure and upper material flexibility has previously been suggested [33].

The clinical implication of our study is that changing the toe‐box width and area of the usual footwear worn by older women with painful, moderate or severe HV may not necessarily reduce medial forefoot pressures, particularly if the footwear has been worn for a long period of time. As such, footwear selection for this group needs to consider all facets of footwear, including duration of wear and upper materials, and not width or area alone. Furthermore, our findings suggest that generic footwear advice should not be the only management provided for HV, but be part of an individualised, multimodal approach including orthotic devices and muscle strengthening/retraining exercises [34].

Our study has several strengths. First, this is the first study to investigate medial forefoot pressures among people with HV using a matrix style device. This reduced the risk of measurement error due to sensor migration and placement error that can occur with discrete sensors [35]. Second, participants were representative of people who most frequently present with HV based on age and sex [2]. Third, we normalised timing to stance phase duration, which has not been done in previous studies. Finally, we investigated medial forefoot pressures using participants' own footwear, which improves the external validity of our findings.

Although this study has several strengths, the findings must be interpreted in the context of its limitations. First, the pedar pad is a novel approach to measuring in‐shoe pressures, but further investigation of the reliability and validity of this system is required. The pedar pad is bulkier than discrete sensors and was difficult to adhere to the foot in participants who wore very tight shoes. Peak pressures may have been underestimated due to the need to zero the device when the shoe is worn, and the pedar pad is only able to document perpendicular forces. Second, our participants were drawn from a pilot and feasibility trial. Consequently, our sample was relatively small, and our results may not be generalisable to the broader population of people with HV. As stated previously, our sample wore shoes that were not overly constrictive, which may explain why we only found weak relationships between toe‐box dimensions and medial forefoot pressures. Third, we did not consider the flexibility of the deformity, and it is possible that pressures may vary depending on the available range of motion of the first metatarsophalangeal and interphalangeal joints. Fourth, hand tracing of footwear dimensions may not have optimal accuracy compared to three‐dimensional foot scanning. Finally, as this is a cross‐sectional study, causal relationships cannot be inferred.

Further research is required to better understand the relationship between toe‐box dimensions and medial forefoot pressures in HV. Investigating other methods for measuring footwear characteristics such as thickness and the pliability of the upper may yield valuable insights. Future studies would benefit from larger sample sizes and a greater variety of age groups. Of particular interest would be younger women, as women with HV are more likely to report wearing footwear with a constrictive toe box between the ages of 20 and 39 years [11]. In addition, comparison of people with various stages of HV may provide insights into how HV severity modulates in‐shoe medial forefoot pressures. Furthermore, longitudinal studies may expand our understanding of the temporal relationship between footwear shape, medial forefoot pressures and HV development.

In conclusion, our study demonstrated that peak pressure and maximum force were greater and peaked slightly later during late stance phase at the distal forefoot compared to the proximal forefoot during level walking in women with moderate or severe painful HV. Notably, there were no statistically significant relationships between toe‐box shape and medial forefoot pressures during walking. These findings demonstrate that older women with painful HV may self‐select footwear with appropriate toe‐box fit and shape to accommodate their deformity, and therefore management recommendations for HV should include tailored multimodal approaches, beyond education to wear wider fitting footwear.

## Author Contributions

**Katrina J. Bajraszewski:** data curation, formal analysis; writing–original draft. **Polly Q. X. Lim:** data curation, writing–review and editing. **Andrew K. Buldt:** conceptualisation, funding acquisition, writing–review and editing. **Sheree E. Hurn:** conceptualisation, funding acquisition, writing–review and editing. **Karen J. Mickle:** conceptualisation, funding acquisition, data curation, writing–review and editing. **Edward Roddy:** conceptualisation, funding acquisition, writing–review and editing. **Anita E. Wluka:** conceptualisation, funding acquisition, writing–review and editing. **Bircan Erbas:** conceptualisation, funding acquisition, writing–review and editing. **Shannon E. Munteanu:** conceptualisation, funding acquisition, methodology, formal analysis, writing–review and editing, supervision. **Hylton B. Menz:** conceptualisation, project administration, funding acquisition, methodology, formal analysis, writing–review and editing, supervision.

## Ethics Statement

Ethical approval was granted by the La Trobe University Human Ethics Committee (reference HEC20474).

## Consent

Informed written consent was obtained from all participants.

## Conflicts of Interest

The authors declare no conflicts of interest.

## Data Availability

Unidentified data from this paper are available from the corresponding author upon reasonable request.
